# Supplementary figures and images for: A high-throughput whole cell screen to identify inhibitors of Mycobacterium tuberculosis
Source: PLoS One. 2019 Jan 16;14(1):e0205479. doi: 10.1371/journal.pone.0205479 (PMC6334966; doi:10.1371/journal.pone.0205479)

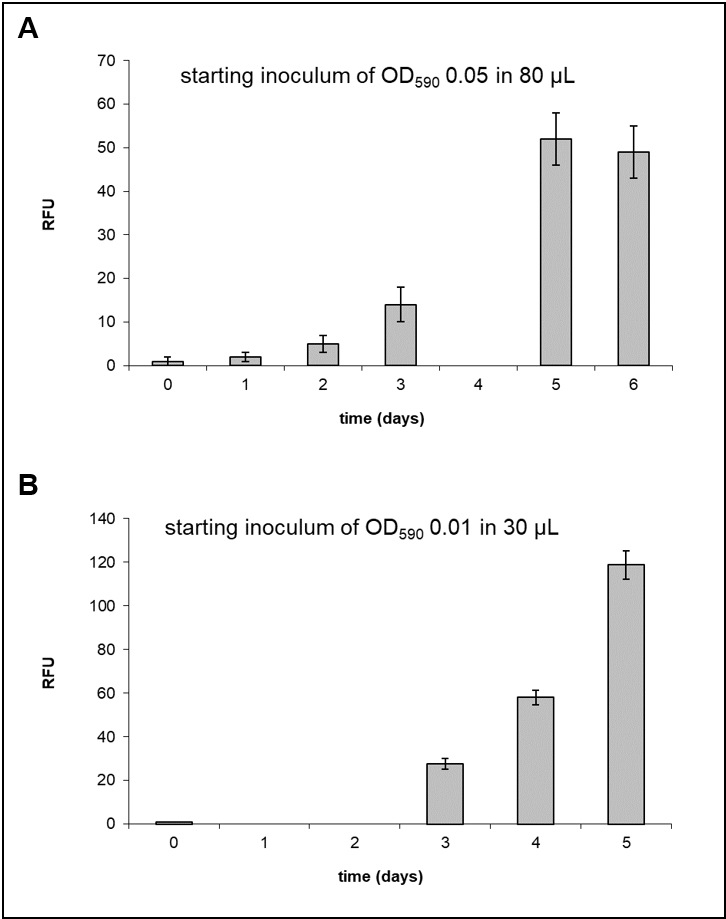

Supplement: S1 Fig — (A) CHEAM3 was diluted to an OD590 of 0.05 and 80 μL was plated in the wells of 384-well plates and incubated for 1, 2, 3, 5 or 6 days. Fluorescence was measured and the mean fluorescence from 192 wells (12 columns) is plotted along with error bars indicating the standard deviation (B) CHEAM3 was diluted to a theoretical OD590 of 0.01 and 30 μL was plated in the wells of 384 well plates. Plates were incubated for 3, 4 or 5 days. Fluorescence was measured in each well and the average fluorescence (n = 384) was plotted with error bars indicating the standard deviation. (TIF) [file pone.0205479.s001.tif]
